# The Impacts of Vaping on the Cardiovascular System: A Systematic Review of Case Reports

**DOI:** 10.7759/cureus.77780

**Published:** 2025-01-21

**Authors:** Stephanie Nagy, Aisha Abdool, Olivia Rocco, Noor Shirazi, David Israilov, Nabiha Atiquzzaman, Kayvan Amini, Marc M Kesselman

**Affiliations:** 1 Rheumatology, Nova Southeastern University Dr. Kiran C. Patel College of Osteopathic Medicine, Davie, USA; 2 Rheumatology, Nova Southeastern University Dr. Kiran C. Patel College of Osteopathic Medicine, Fort Lauderdale, USA; 3 Cardiology, Nova Southeastern University Dr. Kiran C. Patel College of Osteopathic Medicine, Davie, USA

**Keywords:** arrhythmias, cardiac arrest, cardiomegaly, cardiovascular disease, e-cigarettes, electronic cigarettes, juul, myocardial infarctions, pericarditis, vaping

## Abstract

Currently, cigarette smoking remains a global epidemic, with approximately one billion tobacco smokers worldwide, despite declines in use. Vaping products have become popular alternatives in recent years to conventional cigarettes. There has been a perception that vaping serves as a "healthier" alternative and has been increasing across all age groups, especially among teenagers and young adults. Vaping and its additives have been shown to have various implications across organ systems, including pulmonary and cardiovascular systems. Meanwhile, additional research efforts are needed to gain insight into the pathophysiology of vaping on cardiovascular disease onset, progression, and outcomes. In order to better understand the body of literature available on the association between vaping and cardiovascular function, a systematic review was conducted, which included case studies of patients engaged in vaping at the time of developing a cardiovascular event. A systematic review was conducted with a total of 20 patients. Only case studies were included to assess the cardiovascular outcomes that were reported with vaping using either nicotine, non-nicotine, or tetrahydrocannabinol (THC)-containing products. Patients were excluded if they orally consumed the vaping liquid and did not smoke the product. Fourteen of the 20 patients assessed were males (70%), five females (25%), and one unspecified sex (5%). The patients varied in age from 13 to 70 years of age, with a mean age of 27.6 years. Vaping history duration ranged from two days to eight years, with a mean of 22.8 months. Fourteen of the 20 patients reported using nicotine-containing vape devices, with an additional two using THC-containing vape products, three using both nicotine and THC-containing vape devices, and one non-nicotine-containing vape device. The results demonstrated that eight of 20 patients presented with chest pain or discomfort and 10 were found in sudden cardiac arrest. Upon resuscitation from cardiac arrest, a variety of arrhythmias were seen, including ventricular tachycardia, ventricular fibrillation, polymorphic ventricular tachycardia, atrial fibrillation, and a prolonged QT segment arrhythmia. The results suggest that vaping likely negatively impacts the cardiovascular system. Further research is warranted, especially as the popularity of vaping continues to rise among younger populations. As vaping continues to rise in popularity, particularly among younger populations, further research is warranted to elucidate its long-term cardiovascular impact.

## Introduction and background

Currently, cigarette smoking remains a global epidemic, with approximately one billion tobacco smokers worldwide and 5.5 trillion cigarettes produced annually, despite declines in use [1]. Cigarettes are the most common form of tobacco consumption, but other products are widely used, including pipes, cigars, and “smokeless” forms of chewed or sniffed tobacco [2]. In addition, electronic nicotine delivery systems (ENDS), commonly referred to as vaping products, have become popular alternatives in recent years to conventional cigarettes. Vaping has increased across all age groups, especially among teenagers and young adults, because it is viewed as a "healthier" alternative. Vaping is most prevalent among younger, non-minority smokers and those in higher socioeconomic statuses [3,4]. The National Youth Tobacco Survey found that, from 2011 to 2018, the number of high school students using ENDS increased from 1.5% to 20.8% and that middle school users increased from 0.6% to 4.9% [5,6]. In the following year, more than four million high school students (27.5%) reported ENDS use compared to 5.1% using cigarettes [6]. A study comparing vaping trends in the United States, Canada, and England between 2017 and 2022 and found a similar increasing trend within all three countries. Youths vaping were found to have substantially higher dependence rates, higher craving levels, and an increase in the number of days vaping monthly [7]. The uptake in ENDS is due to them being perceived as less harmful than traditional cigarettes. Additionally, 79.8% of users reported switching to vapes due to their perceived reduced harm, 75.4% used them to help reduce their cigarette smoking, and 85.1% stated they used them to quit smoking [4].

Cigarette smoking is associated with severe health implications, with smoking-related deaths largely the result of lung cancer, coronary heart disease, and chronic obstructive pulmonary disease. Smoking also increases the risk of blindness, deafness, back pain, osteoporosis, stroke, and peripheral vascular disease. The respiratory system is conventionally thought to be the most impacted by smoking, but the cardiovascular system has been shown to be equally impacted, even at low levels of smoking exposure. Smoking results in widespread inflammation throughout the body. This can lead to the development of atherosclerosis, reduction in the oxygen supply to myocardial tissues, and limited blood supply to the heart due to vasoconstriction and endothelial dysfunction [3,8].

Vaping has been cited as a new public health crisis [9]. The aerosols produced by vaping contain harmful toxins and carcinogens similar to cigarette smoking that lead to pulmonary exacerbations, cardiovascular disease, and systemic inflammation [10,11]. In particular, individuals who use vaping products have decreased heart rate variability, indicating a shift towards sympathetic dominance while disrupting the renin-angiotensin-aldosterone system. This increases the risk of heart attack and sudden cardiac death. Vaping also has been shown to elevate oxidative stress and inflammation, identical to traditional tobacco cigarettes [12]. In addition, it has been found to significantly increase hyperemia and arterial stiffness while decreasing peak expiratory flow [13]. When comparing cardiovascular outcomes between vaping and cigarette use, no improvements were found in reducing cardiovascular risk including stroke, myocardial infarction, or coronary heart disease [14,15].

Vaping has been shown to be associated with a variety of systemic implications, mostly through alterations of cellular function. For example, chronic vaping has been shown to result in an increased IL-6 pathway, a key cytokine in the inflammatory pathway, which also plays a direct role in progressing plaque buildup and atherosclerosis leading to stroke, myocardial infarction, coronary artery disease, and peripheral artery disease [16,17]. Specific downstream effects of the IL-6 pathway include increased platelet microparticles, which are key in the development of thrombotic conditions [18]. In addition, vaping can lead to significantly increased levels of endothelial progenitor cells, which are found within chronically inflamed tissue and are tied to vasculogenesis [19-21]. These immunologic pathways play a critical role in initiating the acute phase response, which is the complex series of changes that lead to pro-coagulative and inflammatory effects. Inhaled aerosols from vaping have been shown to contain multiple cytotoxic compounds, including nicotine, formaldehyde, acetaldehyde, and metal particles, which have been shown to have a variety of negative effects on multiple organ systems. The cytotoxic contents of the fluid contained in e-cigarette cartridges were found to be detrimental to pulmonary fibroblasts, epithelial cells, and human stem cells [22].

The culmination of these cellular effects has been tied to impaired cardiovascular function. Specifically, vaping has been shown to significantly impair blood vessel function by increasing their permeability and elevating the number of reactive oxygen species in the form of hydrogen peroxide when compared to both cigarette smokers and non-smokers [23]. The National Institutes of Health found that the risk of heart failure with a preserved ejection fraction increased by 19% in those who vaped; however, those who used both cigarettes and vapes had a 59% elevated risk of heart failure [24].

While there is a large amount of evidence within the literature highlighting the relationship between vaping and lung function, a better understanding of the effects of vaping on additional organ systems, including the cardiovascular system, is warranted. As such, the purpose of this systematic literature review is to summarize the impact of vaping on the cardiovascular system through case reports of cardiac events that occurred with vaping use.

## Review

Methods

Search Strategy

A systematic literature review was performed using CINHAL, OVID, EMBASE, and Web of Science using the search terms "vaping" OR "e-cigarettes" OR "electronic cigarettes" AND "cardiovascular disease" OR "heart attack "OR "cardiac arrest" OR "myocardial infarction" OR "heart failure" OR "atherosclerosis" OR "acute coronary syndrome" OR "pericarditis" OR "cardiovascular consequences." To ensure the recency of the articles and with the rise of vaping products occurring within the last decade, only those published between 2010 and 2024 and available in the English language were assessed. The findings were limited to only include case studies or case series to gain a better understanding of any similarities between patient cases. Due to the novelty of the field, there are very limited long-term studies. The articles were analyzed in a step-wise process, first evaluating the title and abstract for relevancy and then assessing the full-text manuscript. Two reviewers were used to analyze the selected studies and decide on the final articles for inclusion. If a conflict arose, a discussion was used to come to an agreement, and the inclusion of a third reviewer was used to break any ties. The Nova Southeastern University (NSU) library database was used to access databases and full-text articles.

Selection Criteria

**Figure 1:**
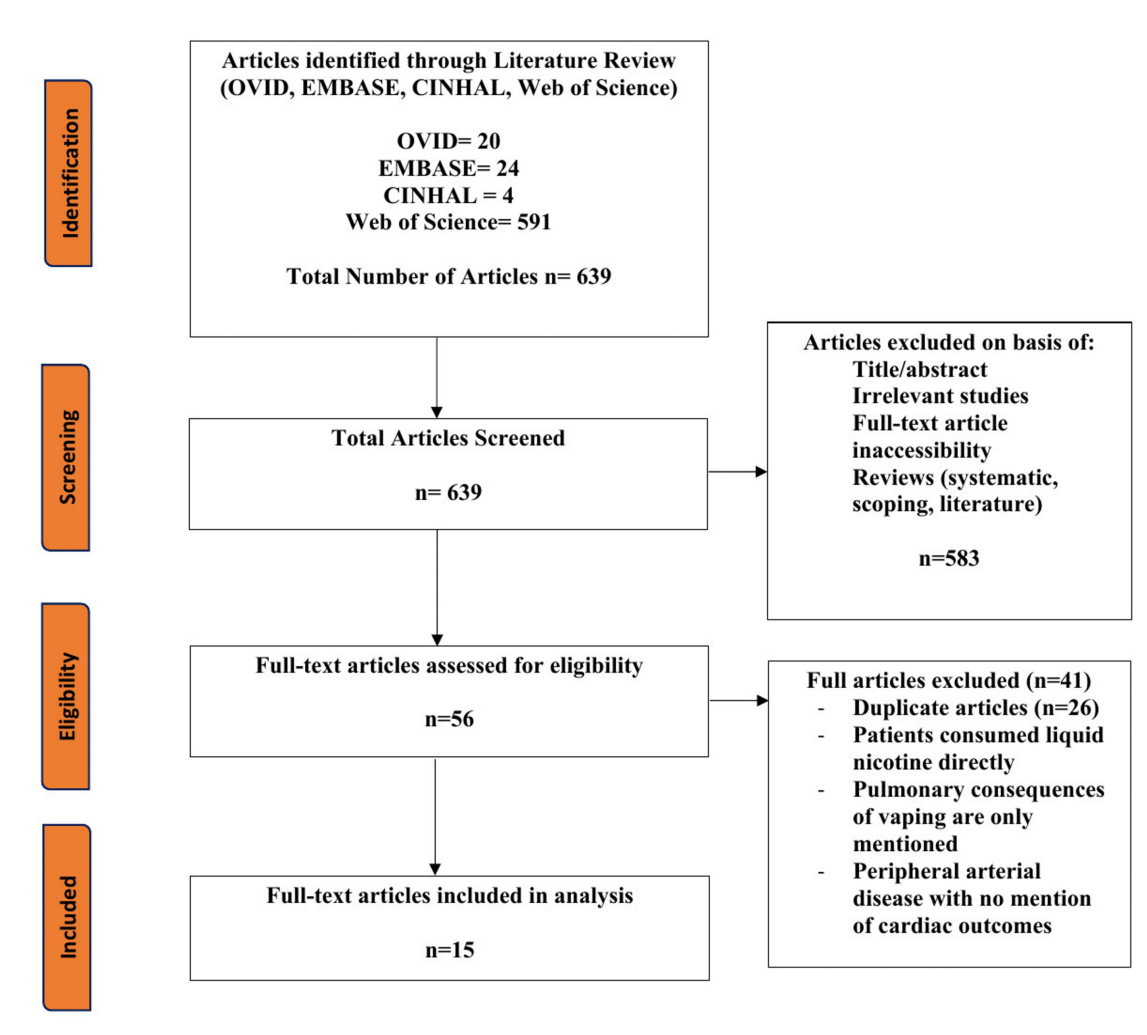
PRISMA flow diagram for the selection of the articles reviewed PRISMA = Preferred Reporting Items for Systematic Reviews and Meta-Analyses

For this systematic literature review, only case studies were included to assess the cardiovascular outcomes that were reported with vaping use of nicotine, non-nicotine, or tetrahydrocannabinol (THC)-containing products. Studies excluded from this review were literature, systematic or scoping reviews, randomized control trials, cross-sectional studies, observational studies, and cohort prospective/retrospective studies. In addition, studies in non-humans, articles published prior to the year 2010, inaccessible full-text versions, articles without English translation, and duplicate studies were removed using Rayyan. To ensure that only patients who inhaled vaping products were analyzed, all patients who directly consumed the liquid nicotine capsules belonging to vaping products for the purpose of suicide were excluded, and any patients who only experienced an isolated lung pathology or did not experience a cardiac event were removed. The preferred reporting items for systematic reviews and meta-analyses (PRISMA) guidelines were followed to enhance transparency and ensure reproducibility of the selection process, as outlined in Figure 1 [25].

Results

In total, 639 articles were populated between the four databases: OVID, EMBASE, Web of Science, and CINHAL. After the first level of screening, 584 articles were removed based on title, abstract, full-text availability, study type, publication year, duplicate articles, and English language availability. This resulted in 55 articles eligible for the second round of screening (in which full-texts were completely screened). Inclusion criteria consisted of case studies or case series, which included patients with a history of vaping and who experienced cardiovascular issues. After analysis and duplicate removal, 15 articles were included in the final review.

**Figure 2:**
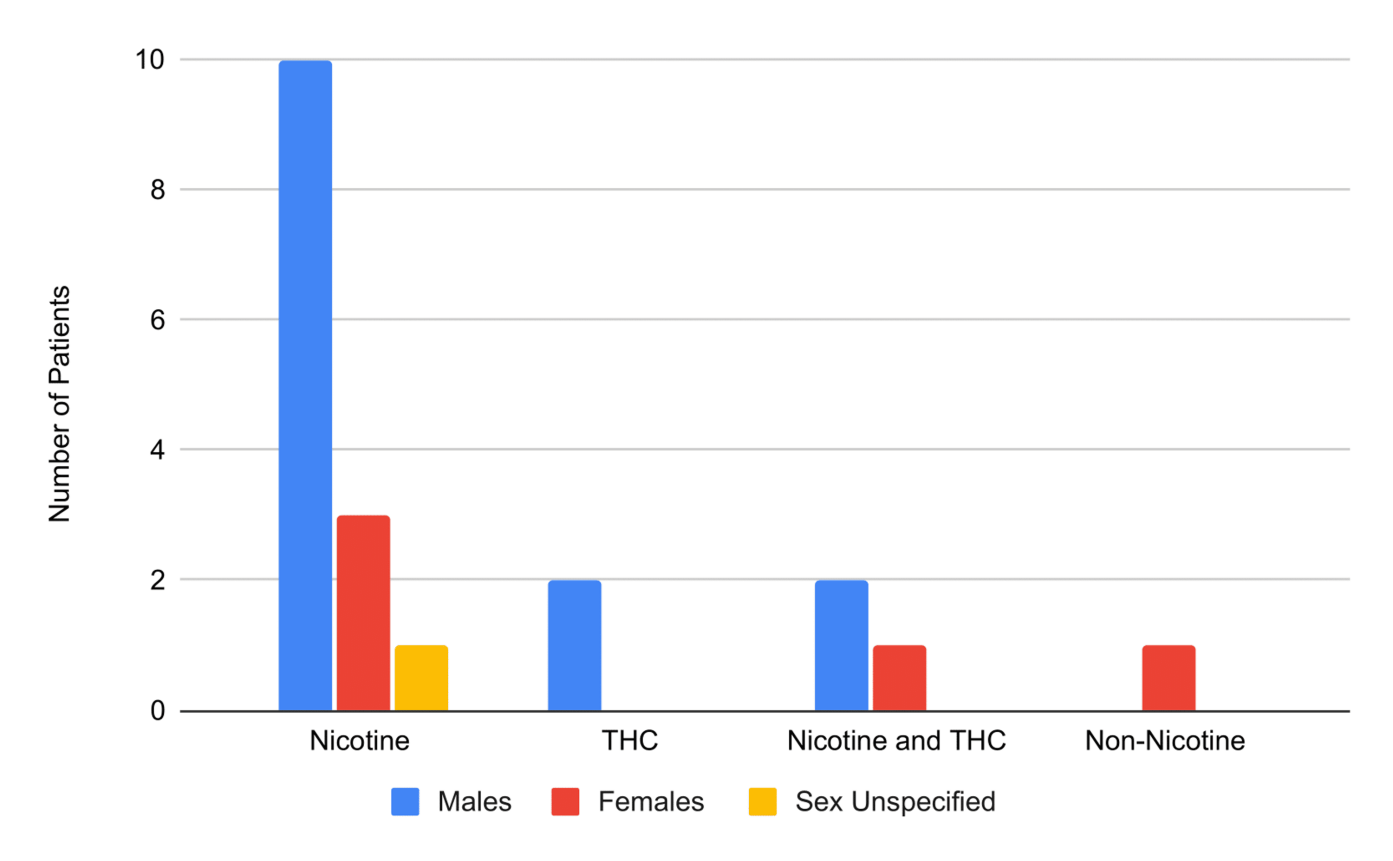
Types of vaping products used in male vs. female patients

**Figure 3:**
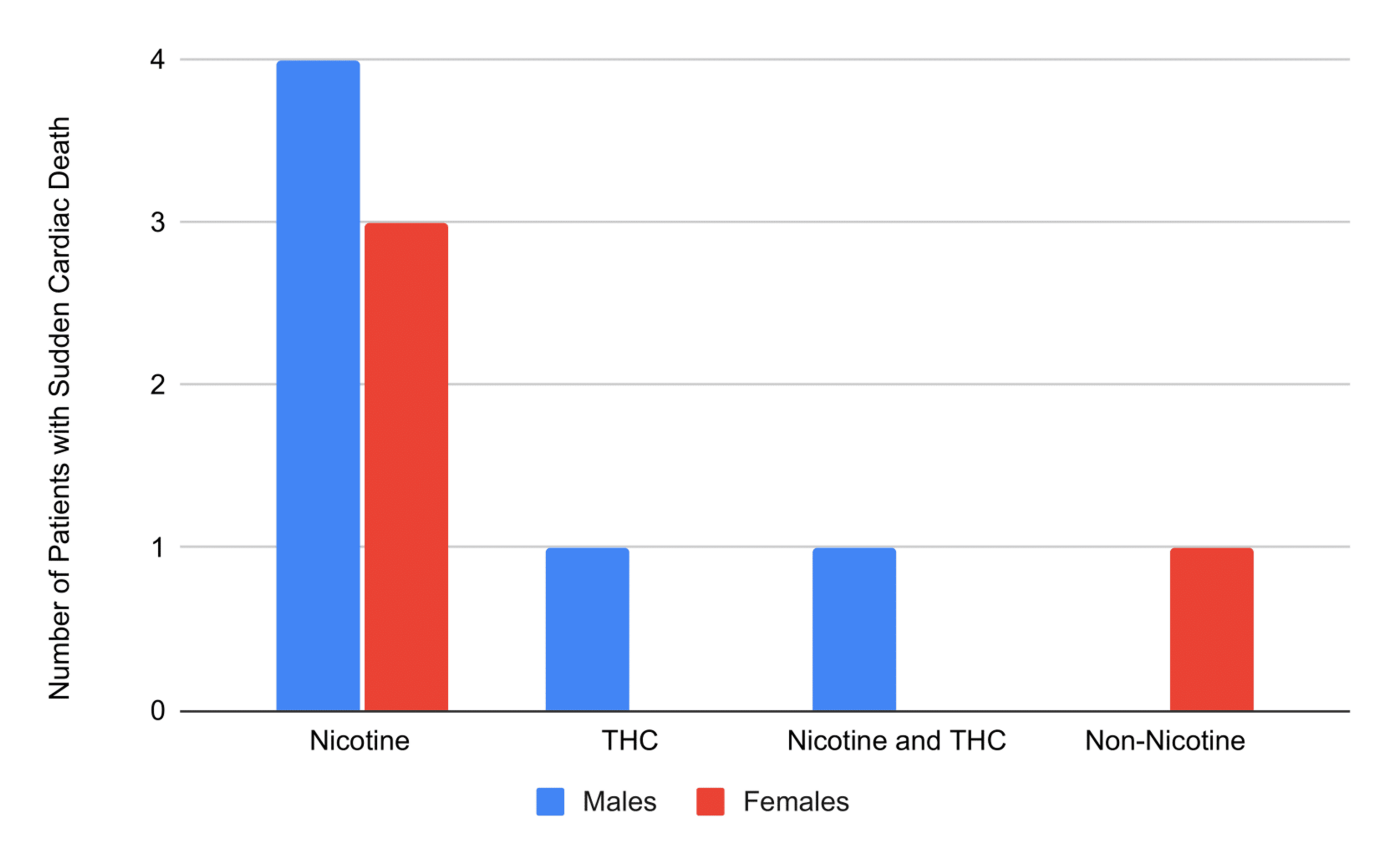
Vaping-related sudden cardiac deaths by sex and type of vapes

Table 1 depicts the studies analyzed, including the number of patients, average age, type and duration of vape use, and patients’ health outcomes. In total, 20 patients were included in the review, with 14 males (70%), five females (25%), and one unspecified sex (5%); this follows the general trend of increased vaping seen in males. The patients varied in age from 13 to 70 years old, with a mean age of 27.6 years. Twelve patients reported their exact vaping history duration, ranging from two days to eight years, with a mean of 22.8 months. A majority of patients reported using nicotine-containing vape devices (14), with an additional two patients using THC-containing vape, three using both nicotine and THC-containing vape, and one non-nicotine-containing vape user (Figure 2). Six patients also reported a history of risky behaviors, such as heavy drinking, anabolic steroid usage, and/or recreational prescription drug use. Eight patients had chest pain or discomfort as a part of their initial presenting complaint. Ten of the 20 patients found in sudden cardiac arrest (Figure 3). Upon resuscitation, a variety of arrhythmias were seen; four were found with ventricular tachycardia, four with ventricular fibrillation, three with polymorphic ventricular tachycardia, one with atrial fibrillation, one with type 1 Brugada, and one with a prolonged QT segment arrhythmias. Following 18 months of vaping abstinence, the type 1 Brugada pattern dissipated in that patient. New-onset arrhythmia persisting after initial presentation was found in one additional patient unrelated to cardiac arrest. This patient had a junctional escape rhythm that was temporally related to nicotine vape use. Four patients had a myocardial infarction, and of those, two patients subsequently died. Two patients had pericarditis, with only one also having a documented lung pathology. One patient had reversible cardiomyopathy. Genetic testing was done on six patients, with clinically significant findings in two individuals indicating positive results for a type 1 LQTS in one patient and a positive result for type 1 catecholaminergic polymorphic ventricular tachycardia in the second patient.

**Table 1:** Characteristics of the patients analyzed in the case studies WBC = White blood count; CRP = C-reactive protein; ESR = Erythrocyte sedimentation rate; NT-proBNP = N-terminal prohormone of brain natriuretic peptide; THC = Tetrahydrocannabinol; VWF = Von Willebrand factor; SpO_2 _= Oxygen saturation; PT = Prothrombin; INR = International normalized ratio; STEMI = ST elevation myocardial infarction; Type 1 LQTS = Type 1 long QT syndrome

Title	Year	Number of patients (M/F)	Age	Reason for seeking medical care	Duration of vaping/e-cigarette use	Cardiac outcome	Lab values	Additional health outcomes
Murphy et al. [26]	2021	1 F	20	Persistent intermittent symptomatic palpitations that occur following vaping use	Not mentioned	Junctional escape rhythm	Lab values all normal	Not reported
Tran Duc et al. [27]	2023	1 M	27	2 days of chest pain	Used a electronic cigarette 2 days before symptom onset	Diffuse ST-segment elevation in leads I, II, aVL, aVF, and V2 through V6, Lead II, V2 - V5 downward-sloping TP segment, Depression in ST segment of aVR, Acutely inflamed pericardium	Temperature 36.7°C, Blood pressure 123/78 mmHg, Heart rate 85 bpm, Respiratory rate 17 bpm, 02 Stat 97%, WBC 15.57 g/L, CRP 10.7mg/dL, Troponin T at admission 5.11 pg/mL, NT-proBNP 5.17 pmol/L	Not reported
Abusharekh et al. [28]	2021	1 M	37	Retrosternal chest pain radiating down the left arm and shortness of breath	4-month e-cigarette use following 10 pack years of cigarette use	Sinus rhythm with ST segment elevation on leads II, III, aVF, and V6 with reciprocal depression on leads V1-V5., Total occlusion of proximal potion of the high diagonal artery, Hypokinesia in the mid-basal segment of the anterolateral wall, Left ventricular ejection fraction <50%. Cardiomegaly	WBC 14.5 (× 10^3^/mL), Neutrophils 82.6%, Eosinophils 0.3%, Lymphocytes 9.4%, Monocytes 7.4%, Hemoglobin 14.9 gm/dL, Platelet 262,000/mL, ESR 48 mm, CRP 31.3 mg/L	Blunting of the right costophrenic sinus. Loss of diaphragmatic contour in the right lower lung zone. Bilateral pleural effusion. Peribroncovascular thickening bilaterally. Ground-glass infiltrates in the lower lobes of both lungs and posterior of the upper lobes bilaterally
McMillan et al. [29]	2023	1 M	17	Acute chest pain, Respiratory distress	Nicotine and THC-containing e-cigarettes for 3 years	Right heart strain, Biventricular dysfunction	D-dimer 16,445 ng/mL	Extensive bilateral pulmonary embolism, pulmonary hypertension, Elevated factor 8 and VWF
Noble et al. [30]	2017	1 M	70	Presented 1 week prior with chest pain, exertional dyspnea and duodenal ulcers. Non-ischemic electrocardiogram and negative serial troponin measurements. Following blood transfusion the chest pain and dyspnea dissipated	Jack Herer Cannabis Extract, THC 64.75%, CBD 1.24% 1 week	Pulseless electrical cardiac activity multiple times, acute myocardial infarction of the anterior left ventricular wall, acute thrombosis of the left anterior descending artery, near complete occlusion of the right coronary artery, and atherosclerotic disease of multiple coronary arteries	Not reported	Not reported
Brennan et al. [31]	2023	1 M	35	Lower back pain radiating down the thighs, fatigue, shortness of breath and chest tightness, Symptoms occurred following strenuous activity of pushing a car with increased use of vape	Vaping pen	S1Q3T3 pattern on ECG due to a right ventricular strain with a absence of pulmonary embolism, Dilated left ventrical, Reduced systolic function with a ejection fraction of 32%, Diffuse hypokinesis, Left ventricular end diastolic pressure of 23mmHg, ECG taken prior to admission had no abnormalities	SpO2 85%, NT-proBNP 28,900 pg/mL, Troponin I 0.271 ng/mL, CRP 14.10 mg/dL, ESR 94 mm/hour	Bilateral pulmonary edema, Right lung consolidation. Prior MVA with ascending and descending aortic pseudoaneurysm, partial aortic transection with subsequent thoracic endovascular aortic repair (TEVAR), and lung and liver laceration after being struck by a motor vehicle – the timeframe between accident and cardiac presentation was not indicated. Patient – admitted to using creatine, testosterone, and methasterone for bodybuilding
Sayeed et al. [32]	2020	1 M	18	Found unresponsive	Vaping	Ventricular fibrillation	Not reported	Bilateral pulmonary infiltration
McClelland et al. [33]	2021	1 M	21	6 years of vaping. Increasing usage, at time of presentation refilled vape 25 times a day with 3mg of nicotine. The switched to JUUL due to higher nicotine content. Estimated every two days the patient inhaled 40-50mg of nicotine. The patient also began to use tetrahydrocannabinol (THC) infused vape cartridge with over 85% THC	Vaping	Inverted T-waves in inferior, lateral and V3 leads, Right axis deviation, Dilated cardiomegaly, Global hypokinesis, Left ventricular ejection fraction 30-35%, Right ventricular enlarged, Decreased right ventricular function, Valve regurgitation Left bundle branch block. Readmission: Ejection fraction of 50%, pericarditis, inflammatory heart disease	Magnesium 1.5 mg, Albumin 2.5 mg, Blood pressure 148/102, Heart rate 120, SpO2 86%, PT 18, INR 1.6	Hypothesized that vaping with substance use resulted in respiratory virus leading to pulmonary collapse and migration to the pericardium resulting in pericarditis, Pleural effusion, Atelectasis
Ahmed et al. [34]	2024	1 F	22	Presented unresponsive with cardiac arrest	1 year of vaping non-nicotine e-cigarettes	During resuscitation patient transitioned from ventricular tachycardia to ventricular fibrillation to atrial fibrillation	ESR 1 mm/h, CRP 3.22 mg/L	Sudden cardiac arrest work up unrevealing, External defibrillation vest revealed no further arrhythmias
Ali et al. [35]	2021	1 M	47	Patient experienced a recent vaping-associated pulmonary injury then they presented with acute anginal chest pain, diagnostic workup was nondiagnostic. Patient returned a week later with a STEMI	Vaping	STEMI, Total occlusion of the proximal left anterior descending artery	Not reported	Vaping-associated pulmonary injury prior to presentation
Bains et al. [36]	2024	6 (3 M, 3 F)	Case 1: 19 M, Case 2: 19 M, Case 3:21 F, Case 4:31 F, Case 5: 19 F, Case 6:26 M	Case 1: sudden cardiac arrest 40 minutes after vaping. Case 2: sudden cardiac arrest a few hours after vaping. Case 3: sudden cardiac arrest following her second time vaping, the patient experienced heavy breathing and collapsed. Case 4: sudden cardiac arrest. Case 5: found deceased. Case 6: dyspnea and diagnosed with vaping-associated lung injury. Released from hospital, then that evening experienced a sudden cardiac arrest	Case 1: 3 weeks of vaping. Case 2: 1 year of vaping. Case 3: “occasionally” vaping. Case 4: More than 1 year of vaping. Case 5: 10 weeks of vaping. Case 6: 3 months of vaping, nicotine and THC products	Case 1: Sudden cardiac arrest to ventricular tachycardia, diagnosed with idiopathic ventricular fibrillation. Case 2: cardiac arrest to ventricular fibrillation. Case 4: 1 week before the event the patient went to the ER with prolonged QT. During current admission the patient experience ventricular tachycardia. Case 5: Found deceased. Case 6: Focal hemorrhage in the interventricular septum	Case 4: hypomagnesemia, Hypokalemia	Case 3: Patient has type 1 catecholaminergic polymorphic ventricular tachycardia but, has been event free for 3 years. Patient was also consuming energy drinks prior to her cardiac arrest. Case 4: Type 1 LQTS genotype-positive but, phenotype-negative. Patient also experience alcohol intoxication. Case 5: Bronchial tree along with eosinophilic infiltrates in the lungs. Case 6: Acute respiratory distress syndrome, pulmonary edema
Fernandez et al. [37]	2024	1 M	22	1-week history of productive cough, hemoptysis, fever, and vomiting. 2-day history of history severe chest pain with dyspnea, diaphoresis, and myalgia	2-year history of vaping	ST‐segment elevation in leads V1‐V4, Dilated left ventricle, Hypokinesia of the basal to mid‐interventricular septum and anterior wall, Depressed overall systolic function, Chronic total obstruction of the mid‐Left anterior descending and Right coronary artery	Heart rate 111 bpm, Blood pressure 98/64 mmHg, Respiratory rate 24 bpm, Temperature 36.6°C, SpO2 87%, Troponins 45,440 pg/mL, WBC 17.2 × 10^3^/mL, Neutrophils 79%, Haemoglobin 15.1 gm/dL, Platelet count 348,000/mL	Bibasilar crackles, Consolidation on the right upper and middle lung fields
Seri et al. [38]	2022	1 M	48	Developed a cardiac arrest at home	THC vaping for 8 years	Polymorphic ventricular tachycardia, Type-1 Brugada pattern	Blood pressure 150/130 mmHg, Heart rate 120 bpm, Respiratory rate 23 bpm, SpO2 95%, Elevated troponin-T level	No gene mutations associated with Type 1 Brugada presentation. Following 18 months abstinence no Type 1 Brugada pattern not present
Glenski et al. [39]	2021	1 M	13	Developed heaviness in the chest and lightheadedness then collapsed at school, patient was vaping throughoutthe day and prior to collapse	Nicotine containing vape	Heart rhythm alternated between ventricular fibrillation and polymorphic ventricular tachycardia, Anomalous left coronary artery originating from the right sinus of Valsalva		
Amirahmadi et al. [40]	2021	1 F	19	Presented with productive, non-bloody cough, dyspnea, sinus and throat irritation, dizziness and headache. Patient was admitted 1 week prior with progressive dyspnea and productive cough	Vaping	Non-specific ST segment depressions in V1 and V2, T wave inversions in AVL, V3 and prolonged QTc	Lactate dehydrogenase 1817 μ/L, Haptoglobin 362 md/dL, D-dimer tests 3360 ng/mL, Troponin I 0.415 ng/mL, NT-proBNP 3750 pg/mL	Respiratory distress, with supraclavicular and intercostal retractions

Discussion

The increasing use of vaping products has grown as a public health concern in recent years [9]. While vaping was initially marketed as a safer alternative for traditional smokers to assist them in quitting cigarettes, it has become popular among many groups, especially to a younger generation of individuals. This is attributable to the variety of flavored e-liquids and stylish portable devices available on the market [9]. While there has been much exploration and documentation of the pulmonary risks associated with vaping, such as acute lung injury and chronic respiratory conditions, the cardiovascular effects of vaping have yet to be fully elucidated. The main compound in the aerosol produced by vaping in most cases is nicotine, which has been shown to be associated with negative cardiovascular outcomes [41]. Vaping has been perceived by the public to be “healthier” than traditional cigarettes, and this could not be further from the truth. A common brand offering these products is JUUL. One JUUL pod contains 28.8 mg of nicotine, which is the same amount of nicotine as a pack of cigarettes [42]. This indicates a larger focus on educating about the risks of vaping.

Nicotine binds nicotinic receptors in the heart, which causes an increase in cardiac work, cardiac contractility, blood pressure, and heart rate. As a result of increased heart rate, cardiac output increases as well. Peripherally, the interaction between nicotine and the nicotinic receptor causes systemic vasoconstriction, resulting in reduced blood flow to organ systems. The peripheral vascular system is not excluded from the effects of nicotine use either. Nicotine is thought to have a role in thrombogenesis and platelet activation [43]. Acute exposure to vaping can contribute to arterial stiffness, a major contributor to thrombosis-related cardiovascular disease [44]. A single use of an e-cigarette can cause an increase in 8-iso-prostaglandin, a marker for oxidative damage that is inversely correlated with nitric oxide levels, indicating endothelial dysfunction. The lack of nitric oxide levels allows for the production of pro-inflammatory markers in blood vessels, attracting monocytes and other phagocytic cells and inducing endothelial and subendothelial damage [45]. Additionally, intercellular adhesion molecule-1 was significantly elevated, indicating the potential for endothelial dysfunction and pro-inflammatory processes due to vaping [46].

In the articles reviewed, 10 out of 20 of the patients were found in cardiac arrest. Upon resuscitation, a variety of arrhythmias were documented, including ventricular tachycardia, ventricular fibrillation, polymorphic ventricular tachycardia, atrial fibrillation, and prolonged QT segment arrhythmias. A potential reason behind these outcomes is the impact that nicotine has on activating the sympathetic system, resulting in myocardial remodeling, often manifesting clinically as arrhythmias and ventricular dysfunction [47]. A novel study compared left ventricular function in patients who smoked cigarettes, vaped with nicotine-containing products, and vaped with non-nicotine products. Standard cigarettes increased the first intrinsic frequency of the left ventricle, leading to contractility dysfunction, while vaping products with nicotine decreased the second intrinsic frequency of the left ventricle, leading to reduced vascular supply. Interestingly, those who vaped non-nicotine products had no significant difference from the controls. Nicotine-containing vapes resulted in a significantly reduced function of the left ventricular compared to non-nicotine-containing vapes [48]. Therefore, further results must be conducted on analyzing the impact of nicotine, THC, and non-nicotine vaping products on the occurrence of cardiac arrest and subsequent arrhythmia.

Within the reviewed case studies, half the patients had some form of comorbid lung pathology, including e-cigarette or vaping product use-associated lung injury, bibasilar crackles, consolidation, pleural effusion, atelectasis, pulmonary infiltrates, and one patient experienced a pulmonary embolism leading to pulmonary hypertension and subsequent heart failure [29]. Pulmonary conditions other than those diagnosed with a vaping-associated pulmonary injury are difficult to tie directly to ENDS use; however, they did temporally occur following the initiation of vaping. In cardiopulmonary circulation, the heart is perfused with the contents inhaled by the lungs through the alveoli before any detoxification can be performed by the liver [49,50]. Transfer of toxic metabolites from vaping to the chambers of the heart and even coronary vessels may lead to structural and functional abnormalities, which can progress to arrhythmias and cardiac arrest. Pulmonary embolism leading to cardiac arrest is a rare phenomenon, only accounting for 3% of cardiac arrest cases [36,51]. The link between pulmonary concerns and vaping is evident, suggesting that pulmonary insults may contribute to subsequent negative cardiovascular outcomes.

The presence of nicotine is not the only harmful inhalant tied to vape-derived aerosol. Vaping emits carbonyl compounds from the thermal decomposition of e-liquid ingredients, which include notably formaldehyde, acetaldehyde, acrolein, glyoxal, and propylene glycol [10]. These additives occur in different concentrations due to a lack of regulation. Formaldehyde, acetaldehyde, and glyoxal have been shown to be associated with carcinogenic contributions to vape aerosol. Formaldehyde, classified as a Group 1 human carcinogen, has been conclusively linked to cancer. The others have been categorized as possibly associated with carcinogenic effects [10,52,53]. These additives to vapes have been found to have significant negative impacts on the cardiovascular system. Formaldehydes have been found to cause endothelial dysfunction, elevated levels of oxidative stress, and inflammation, resulting in cardiovascular diseases [54]. In addition, exposure to formaldehyde through vaping aerosols has been shown to result in oxidative and alkylation DNA lesions. The greater the exposure, the greater the damage. Aldehydes have a bimodal effect on aldehyde dehydrogenase 2 (ALDH2), which protects the heart from oxidative damage. At low levels, it has been shown to improve the ability to remove reactive oxygen species. Meanwhile, at high levels, it has been demonstrated to lead to ALDH2 dysfunction, which can cause oxidative damage to the heart [55,56]. Glyoxal has also been shown to induce the expression of cyclooxygenase-2, leading to inflammatory injury of endothelial cells [57]. All these compounds may be found in different concentrations of vaping products depending on the brand and manufacturer as there is no regulation currently in place. Meanwhile, the accumulation of even one of these toxic compounds can cause irreversible damage to cardiovascular function.

In two of the patients, it was found that vaping triggered a previous benign gene mutation (type 1 LQTS and type 1 catecholaminergic polymorphic ventricular tachycardia), resulting in cardiac consequences, following vaping cessation the patients experienced no further cardiac events. Certain cardiac gene mutations may be exacerbated by environmental factors, such as e-cigarette usage. This finding supports the idea that vaping may be particularly dangerous for genetically predisposed individuals and should be explored further. Vaping could have an impact on a variety of genetic variants that have yet to be investigated. For example, a common genetic variant is ALDH2, which is present in 40% of the East Asian population, and the ability to metabolize aldehydes is impaired. This variant has been shown to be strongly associated with atrial fibrillation, hypertension, heart failure, myocardial infarction, and re-perfusion-related ventricular arrhythmias [55,56].

Fatalism is the belief that events are predetermined and, therefore, inevitable. As such, fatalistic beliefs about cancer have been positively correlated with engaging in risky health behaviors, such as cigarette smoking, e-cigarette smoking, and heavy alcohol use [58]. A significant number of patients examined in the study were teens or young adults in their 20s, which is specifically the group that has experienced the greatest increase in vaping uptake. E-cigarette use in high school students has also been linked to other dangerous behaviors such as not wearing a bicycle helmet, texting while driving, current marijuana use, current painkiller use, and current heroin use [58,59]. In addition, risky alcohol use has been linked to an increase in vaping regardless of cigarette history in college-age students [58,59]. Fatalistic beliefs about health may contribute to risky behavior, which has been shown to increase the risk of cardiac events. Six of the 20 patients (30%) included in this data set reported a history of risky health behavior, including substance use, alcoholism, and regular consumption of energy drinks. Anabolic steroid misuse can also be directly responsible for multi-organ dysfunction and has been implicated in multiple cardiovascular cases of death [60]. Overall, steroid usage, substance usage, and excessive alcohol consumption all may have adverse health consequences, which could exacerbate cardiovascular insult, and this area requires further investigation.

Fourteen of the 20 patients included in this study were teenagers or young adults with ages ranging from 13 to 27. E-cigarette advertising often focuses on vaping as a healthier alternative to traditional cigarettes and the positive sensory experience of vaping itself [61]. The Monitoring the Future Annual Report from 2023 supports that individuals perceive vaping as a better alternative to cigarette smoking [62]. They also found record-high use of vaping nicotine or cannabis in the past year among adults aged 19-30 years with a historic upward trend in the past five years. In addition, 19-30-year-old female individuals reported a higher prevalence of past-year cannabis use as compared to male respondents for the first time in 2023 [62].

Major limiting factors of the analysis include a lack of information on each patient. There was limited information available on patients' medical histories. In addition, a large portion of patients had a co-existing lung pathology. Nine of the 20 patients had documented lung pathology because of vaping, with two deaths attributed to a pulmonary cause. This can pose difficulties in pinpointing the exact role vaping played in cardiovascular outcomes. Furthermore, the duration of vaping was commonly omitted, making it difficult to analyze the relationship between the duration of vaping and cardiac outcomes. Most of the patients included in the dataset were teenagers or young adults without a history of cardiac events or monitoring. This population has been historically known to delay seeking adequate care when health concerns arise. As such, some clinical aspects of their vaping use could be non-documented. In addition, the case studies analyzed did not include a complete history of each patient’s vaping history regarding the type of vaping product used. This is significant because nicotine and additive levels vary across products from different manufacturers. Patients varied in the type of vaping liquids, which included documentation of nicotine, THC, and caffeine. Furthermore, with the limitation of the inclusion of only English language studies, there is a potential that additional case reports were missed.

Despite these limitations, the findings underscore the need for further research into vaping's cardiovascular effects. Future studies, including retrospective/prospective cohort studies and case-control studies, could more clearly elucidate the relationship between vaping and cardiovascular events. They should also aim to gain insight into the impact of nicotine-containing products as compared to those without nicotine on cardiovascular insult. Future research exploring autonomic dysfunction, inflammatory response, and atherosclerotic-related pathology among individuals who vape may help elucidate the link between vaping and cardiovascular events.

## Conclusions

Vaping has been shown to be associated with adverse cardiovascular outcomes. While vaping is often promoted as a safer alternative to smoking, the available research to date suggests that vaping can pose significant risks to the cardiovascular system. The inhalation of harmful chemicals, such as nicotine, formaldehyde, and aldehydes, alongside the effects of increased heart rate and blood pressure, can contribute to the development of cardiovascular diseases. It has been shown that individuals who vape commonly experience a variety of negative cardiovascular complications, including arrhythmias and cardiac arrest. In addition, vaping has been shown to trigger underlying genetic cardiac dysfunctions such as type 1 LQTS and type 1 catecholaminergic polymorphic ventricular tachycardia. As the popularity of vaping continues to rise, especially among younger populations, additional long-term large studies are warranted to fully understand the effect of vaping on all body systems. In addition, updated regulations, along with guidance on preventative strategies, will help mitigate the potential cardiovascular harm associated with this growing trend. Stronger education campaigns should be developed on the adverse effects of vaping, in addition to stronger regulation on ingredients, marketing, and availability to purchase vaping devices. As the rate of vaping continues to rise, clinicians should educate patients not only on the negative pulmonary impacts but also on the cardiovascular effects.
